# Mediated amperometry as a prospective method for the investigation of electroporation

**DOI:** 10.1038/s41598-020-76086-2

**Published:** 2020-11-05

**Authors:** Povilas Simonis, Rasa Garjonyte, Arunas Stirke

**Affiliations:** State Research Institute, Center for Physical Sciences and Technology, Saulėtekio al. 3, Vilnius, Lithuania

**Keywords:** Biochemistry, Biophysics, Chemical biology, Microbiology, Chemistry

## Abstract

Pulsed electric field effects induced in a membrane, as well as intracellular structures, depend on cell type, field and media parameters. To achieve desired outcomes, membranes should be permeabilized in a controlled manner, and thus efficiency of electroporation should be investigated in advance. Here, we present a framework for using mediated amperometry as a prospective method for the investigation of electroporation and its effects on cellular machinery. Whole-cell sensors with single mediator systems comprised of hydrophilic or lipophilic mediators were successfully employed to investigate membrane permeability as well as cellular responses. Exposure of yeast cells to single electric field pulse (τ = 300 µs, E = 16 kV/cm) resulted in up to tenfold increase of current strength mediated with hydrophilic mediators. Exposure to PEF resulted in decrease of menadione mediated current strength (from 138 ± 15 to 32 ± 15 nA), which could be completely compensated by supplementing electrolyte with NADH.

## Introduction

In recent years, more and more processes in both medicine and biotechnology depend on the abiotic manipulation of cells. One of the relatively new and prospective techniques used for increasing permeability of cells and tissues is the exposure to pulsed electric fields (PEFs). It is already used for increasing the efficiency of chemotherapy^1^, enhancing gene transfection^2^, extending the shelf-life of food^3^, and improving the extraction of intracellular compounds^4^. One of the main PEF targets in cells is the plasma membrane, which can be substantially and controllably permeabilized (electroporated)^5^ for desired applications. We have shown previously that PEF can induce cell death via metacaspase activation^6^ and permeabilize both cell membranes and yeast cell walls^7^. The experimental data indicated that cellular responses are dependent on electric field strength, pulse duration, and pulse number^8^.

The cellular state after PEF is usually determined by measuring various parameters, including permeability^9^, oxidative state^10^, lipid peroxidation^11^, and other markers related to aging^12^ or death^13^. The measurement of increased molecular transport across the membrane is the most common way to confirm electroporation^14^. The extent of permeabilization can be evaluated by measuring the leakage of intracellular compounds^15^ as well as signals generated by exogenous compounds after entering the cell^16^. To successfully detect permeabilization, exogenous compounds should not enter intact cells and should be easily detectable. On the other hand, some physical methods like measurement of conductivity^17^, impedance^18^, or cell swelling^19^ can assist investigation and detect electroporation without exogenous probes. There is a high number of substances and methods, yet each comes with a set of disadvantages and often indicates only single very specific characteristic or parameter of a system reliably. Furthermore, although PEF is widely applied, electroporation effects on the output signals of various detection methods are poorly understood as most methods are primarily used for analyzing intact cells. Moreover, development of new methods for electroporation detection is very important for understanding the mechanism of electroporation and for investigating the cellular state after exposure to PEF.

Electrochemical methods provide the ability to monitor redox processes inside the intact cells, thus reflecting the intracellular state^20,21^. Such experiments are often conducted by transferring whole cells^22,23^, or their parts^24^ onto surface of an electrode. To achieve the electron transfer between the electrode and redox centers of the enzymes in the cell, an electroactive mediator is used. The mediator shuttles the electrons between the electrode and the redox centers of the enzymes, thus providing information about redox activity of cells. Yet currently electrochemical methods such as potentiometry or amperometry are not widely used for investigating PEF effects on cells.

In this study, we set out to employ amperometry at yeast-modified electrodes to elucidate pulsed electric field effects on (1) plasma membrane/cell wall permeability, (2) metabolic activity, and (3) to investigate the prospects for controlled sensitivity/applicability improvement of whole-cell sensors.

## Materials and methods

### Cultivation and preparation of yeast cells

*S. cerevisiae* BY4742 (*MAT*α; *his*3∆1; *leu*2∆0; lys2∆0; *ura*3∆0) yeast cells (Euroscarf, Germany) were grown on solid media YPD (1% yeast extract (Biocorp, Poland), 2% peptone ex casein (Carl Roth GmbH, Germany), 2% glucose (Merck KGaA, Germany), 1.3% agar (Alfa Aesar, Germany) at 30 °C in the incubator for 48 h. Cells were then collected. 40 mg of cells were washed twice with electroporation buffer (EPB) (20 mmol/L Tris (Applichem, Germany), HCl (Merck KGaA, Germany), pH 7.4), and resuspended in 0.5 ml of it. Conductivity of final suspension was ≈ 1.5 mS/cm (Seven2Go conductivity meter with Inlab 738-ISM sensor, Mettler Toledo, USA). Where noted, such cell suspension was lyophilized and kept at 4 °C before use in experiments.

### Electrode preparation

**Figure 1:**
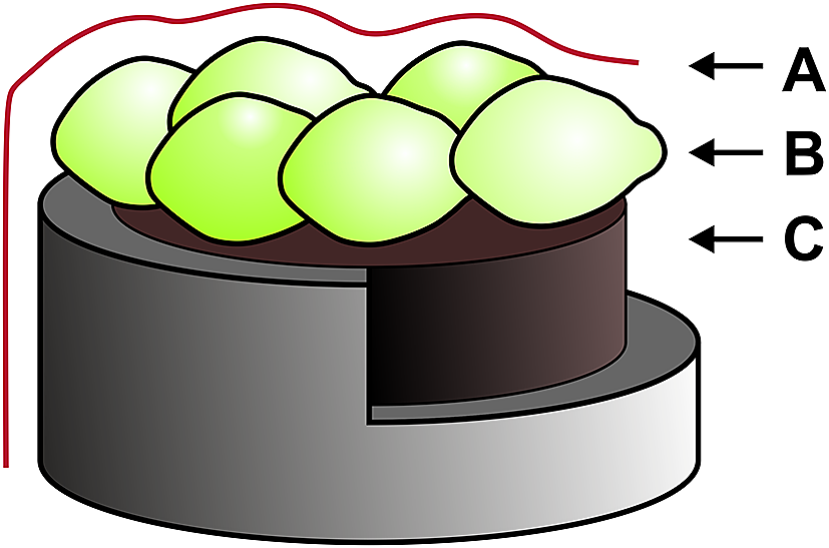
Yeast-modified carbon paste electrode. A membrane; B yeast cells; C carbon paste.

Plain carbon paste was prepared by mixing 100 mg of graphite powder (Fluka, Germany) with 50 µl of paraffin oil (Fluka, Germany)^25^. The paste was packed into an electrode body consisting of a plastic tube (diameter 2.9 mm) and a copper wire serving as a contact for an electrode. The layers of the yeast cells on the surfaces of plain carbon paste electrodes were formed by covering the conducting area with 10 µl of cell suspension in EPB. The electrodes were allowed to dry at room temperature for 15–20 min and were then covered with a dialysis membrane (Sigma-Aldrich, USA) (Fig. 1). Due to relatively long electrode preparation procedure (when compared to membrane resealing time), investigation of permeability is limited to irreversible electroporation.

### Electrochemical measurements

Electrochemical experiments were carried out according to previously described procedure^25^ on a BAS-Epsilon Bioanalytical system (USA) and a three-electrode cell arranged with a magnetic stirrer. The platinum wire and Ag/AgCl in 3 M NaCl served as counter and reference electrodes, respectively. Amperometry was carried out in a stirred solution at an operating potential 0.3 V (vs. Ag/AgCl, 3 M NaCl). The yeast-modified carbon paste electrode served as a working electrode. All electrochemical measurements were performed at room temperature.

#### Hydrophilic mediator

To analyze hydrophilic mediators, yeast cells were employed as biosensors for lactic acid detection^25^. Amperometry for lactic acid-sensing was performed in phosphate buffer: 0.1 M potassium phosphate (Riedel-de Haën, Germany); 0.1 M potassium chloride (Fluka, Germany); potassium hydroxide (Sigma Aldrich, USA) at pH 7.3 and containing 0.5 mM of mediator: *N*-Methylphezonium methyl sulfate (PMS) (Fluka, Germany), 2,6-Dichlorindophenol sodium salt hydrate (DCPIP) (Fluka, Germany), Potassium ferricyanide (PFC) (Fluka, Germany), 1,2-Naphthoquinone-4-sulfonic acid sodium salt (NQSA) (Fluka, Germany). After reaching steady state of the background current at the operating potential (0.3 V), l-lactic acid (Riedel-de Haën, Germany) was added into the solution (final concentration 0.2 mM). Data was collected and represented as a change in current strength after the steady-state current was achieved.

#### Lipophilic mediator

Amperometry for menadione-mediated (Sigma-Aldrich, USA) current detection was performed in phosphate buffer at pH 6.5. The electrode was poised at an operating potential (0.3 V) until the steady-state of the background current was obtained. After that, menadione [dissolved in absolute ethanol (VWR, France)] was added up to final concentration of 67 μM. Change in current was evaluated after the steady-state current was reached. Redox activity monitoring was performed by adding NADH disodium salt (VWR, USA) or NADPH tetrasodium salt (MP Biomedicals, France) to a final concentration of 1 mM.

### PEF generation system

**Figure 2:**
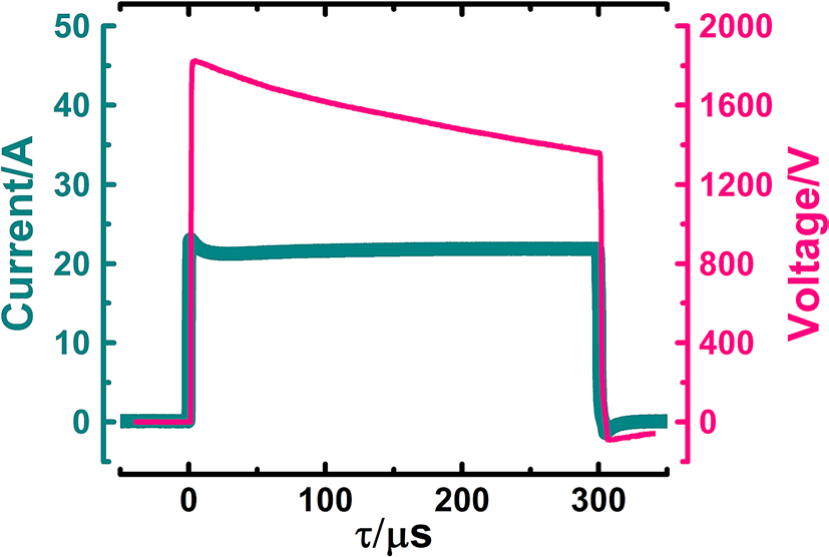
Voltage and current across the cuvette during the generation electric field pulse.

A pulse generator assembled in the Center for Physical Sciences and Technology was used in the experiments^26^. 100 µl of yeast cell suspension was placed into a cuvette with 1 mm gap between electrodes (Fisher Scientific, US) and exposed to a single square-shaped pulse with pulse length of 300 µs and an electric field strength (E) of up to 16 kV/cm (I = 22 ± 0.5 A). Pulses of voltage and current are shown in Fig. 2. Temperature of suspension before and after exposure was evaluated by thermal camera (FLIR One Pro LT, FLIR Systems, US). Electric field pulse raised the temperature of suspension by up to 7 °C.

### Evaluation of the viability and permeability

The viability was evaluated by plating treated cell suspension onto a solid YPD medium and then incubating in the INCU-Line (VWR, USA) incubator at 30 °C for 48–72 h. After incubation, the number of colony-forming units (CFU) was evaluated. As a control, PEF untreated suspension was used.

Permeability of the plasma membrane was measured by employing Sytox Green (Thermo Fisher Scientific, USA). Dye was diluted in anhydrous dimethyl sulfoxide (Thermo Fisher Scientific, USA) to a final concentration of 0.25 mM. During the whole procedure, cells were kept on ice. The solution was transferred to the cell suspension (final concentration—125 nM) one hour after exposure to PEF. After incubation for 60 s, fluorescence intensity was measured using luminescence spectrometer Perkin Elmer LS—50b (PerkinElmer, USA). The excitation wavelength was 480 nm. Emission was measured within range of 500–600 nm with scanning speed of 100 nm/min.

Leakage of intracellular cofactors was measured by exciting supernatant with 340 nm and measuring fluorescence at 430 nm^27^.

## Results and discussion

### PEF effects on yeast viability and membrane permeability

**Figure 3:**
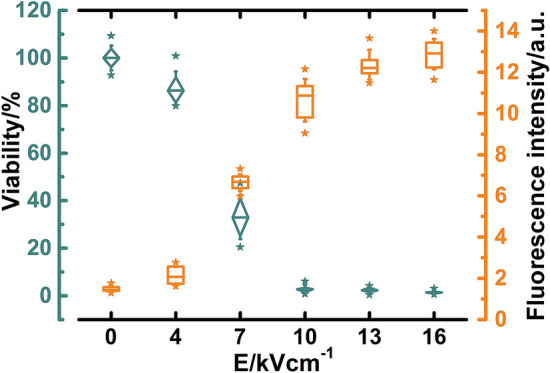
PEF effects on the viability (blue diamonds) and permeability (orange rectangulars) of yeast cells. Whiskers of box plots indicate values of standard deviation while stars beside respective boxes indicate highest and lowest values within the distribution of 10 experiments.

We started the investigation by evaluating viability and permeability of yeast cells with standard methods. Viability was assessed by counting colony-forming units (Fig. 3). After exposure to PEF the viability started to decrease considerably when electric field strengths (E) were higher than 4 kV/cm. After exposing yeast cells to a pulse with E stronger than 10 kV/cm, viability decreased to ~ 2%. Therefore, we concluded that viability decreased due to cellular damage. Only viable cells can replicate and form detectable colonies; thus, we eliminate misinterpretation of cells’ death state^28^. Adjustments in composition of media can also indicate respiration deficiency^29^ and sublethal injuries^30^, but in this study we focused on evaluating the irreversible damage.

Permeability was evaluated with membrane-impermeable Sytox green dye intercalating into nucleic acids. Some cells were permeable to the Sytox green fluorescent dye even before exposure to PEF, possibly due to the non-uniform age of cells^31^ or nonspecific damage during preparation of cell suspension. The rise in membranes’ permeability after the exposure to PEF showed an inverse pattern to the change in the viability. Weak electric field pulses (E < 4 kV/cm) did not change either of the measured parameters. Even if membrane was permeabilized, it effectively resealed without further damage to cells. Most considerable changes were observed when cells were exposed to a pulse with electric field strength between 4 and 10 kV/cm. Stronger electric fields permeabilized yeast cells irreversibly, causing a decrease in the viability, potentially due to the leakage of intracellular compounds. Eukaryotic cells can repair their membrane damage and continue to grow at later times^32^. To avoid misinterpretation of such cases, permeability was measured 1 h after exposure to PEF.

The possibility of using PEF for inactivation of microorganisms is already widely explored and exploited both for research and commercial applications^33^. Full inactivation of the yeast cells was not detected, probably due to differences in cell size, shape^34^ and some volume of suspension being above the electrodes in untreated areas, and non-homogeneous electric field distribution^35^.

Hence, we showed that exposure to a single electric pulse with a duration of 300 µs induces irreversible electroporation and causes a decrease in the viability of yeast cells.

### Yeast pretreatment enhances the signal of current mediated via hydrophilic mediators

One of the factors limiting the usage of hydrophilic mediators in combination with intact cells is permeability of membranes. To test cell pretreatment procedures we chose irreversible electroporation and lyophilization which is typical for the preparation of biosensors^36^. After pretreatment, yeast cells were immobilized on carbon paste electrodes, immersed into the solution with the single hydrophilic mediator, and analyzed.

The system we employed was previously suggested for amperometric biosensing of lactic acid^25^. The scheme for mediated electrocatalytic oxidation of lactic acid can be represented as follows:

$$ \begin{aligned} & {\text{L-lactic}}\,{\text{acid }} + {\text{ Flavocytochrome}}\,{\text{b}}_{{{2}\left( {{\text{ox}}} \right) }} \to {\text{ Pyruvic}}\,{\text{acid }} + {\text{ Flavocytochrome}}\,{\text{b}}_{{{2}({\text{red}})}} \\ & {\text{Flavocytochrome}}\,{\text{b}}_{{{2}\left( {{\text{red}}} \right) }} + {\text{ Mediator}}_{{({\text{ox}})}} \to {\text{ Flavocytochrome}}\,{\text{b}}_{{{2}\left( {{\text{ox}}} \right) }} + {\text{ Mediator}}_{{({\text{red}})}} \\ & {\text{Mediator}}_{{\left( {{\text{red}}} \right) }} \to {\text{ Mediator}}_{{\left( {{\text{ox}}} \right) }} + {\text{ e}}^{ - }.\end{aligned} $$

**Figure 4:**
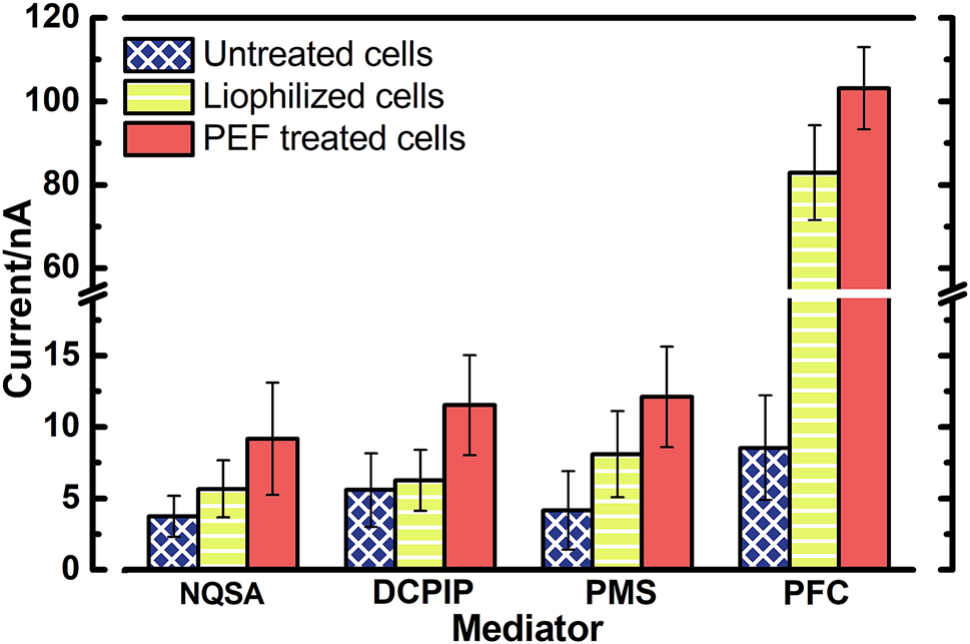
Current dependence on yeast pretreatment and mediator (C = 0.5 mM) used for lactic acid sensing. NQSA—1,2-Naphthoquinone-4-sulfonic acid sodium salt, DCPIP—2,6-Dichloroindophenol sodium salt hydrate, PMS—*N*-Methylphenazonium methyl sulfate, PFC—Potassium ferricyanide. Each mean was obtained from at least 10 experimental data points.

The results showed marked differences in the responses of electrodes covered with untreated, lyophilized, and electroporated yeast cells (Fig. 4). Current generated by PEF treated cells in a solution with ferricyanide was strongest (I = 103 ± 10 nA) followed by lyophilized (I = 83 ± 11 nA) and untreated yeasts (I = 9 ± 4 nA). Exposure to PEF increased permeability to all mediators resulting in stronger currents.

In a similar study, the rise in ferricyanide current strength after chemical pretreatment was most notable as well^37^. It remains to be elucidated whether current strengths generated by NQSA, DCPIP, and PMS during lactic acid-sensing are limited by permeability of the membrane and cell wall or by charge transfer^38^. Since pretreatment by lyophilization and exposure to PEF resulted in generation of stronger currents we suggest that limiting factor is charge transfer and not permeability. Even though lyophilization provides convenient solution for long term storage of yeast cells, the permeability of cell populations is non-homogeneous^39^. PEF treatment was superior to lyophilization, while ferricyanide was shown to be the most efficient mediator and was used for further investigation of the electroporation effects.

### PEF modulates signals of yeast-modified electrodes

**Figure 5:**
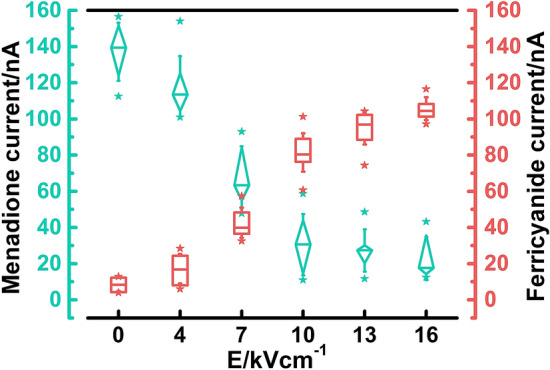
Effect of electric field strength on the current responses to 0.2 mM lactic acid at yeast-modified electrodes at the operating potential 0.3 V in phosphate buffer at pH 7.3 containing 0.5 mM mediator K_3_[Fe (CN)_6_] (pink rectangles), and to 67 μM menadione at an operating potential 0.3 V in phosphate buffer at pH 6.5 (teal diamonds). Whiskers of box plots indicate values of standard deviation while stars beside respective boxes indicate highest and lowest values of distribution.

Usually, redox processes occurring in intact cells are studied using double mediator systems containing both lipophilic and hydrophilic mediators^40,41^. The lipophilic mediator can penetrate membrane and interact with intracellular redox centers while hydrophilic mediator facilitates transferring charge from cell to electrode surface. To note the changes in measured current at yeast modified electrodes we pretreated cells with different electric field strength and tested them with common mediators from double mediator system separately (Fig. 5).

After exposure to weak electric fields (E ≤ 4 kV/cm) measured current mediated with PFC slightly increased from 9 ± 4 up to 17 ± 8 nA. The further rise in electric field resulted in ever-increasing currents. Yet after exposure to pulses stronger than 10 kV/cm, increments of rise in current strength decreased. Such a profile could imply that with the increase in E, more yeast cells are permeabilized irreversibly, and the ever-smaller fraction of cells remain untreated/unpermeabilized. These results indicate that PEF treatment can be used to circumvent the usage of double mediator systems and increase signal mediated by hydrophilic mediators in a controlled manner. After the pretreatment, hydrophilic mediators can efficiently enter cells and participate in electron transfer from intracellular enzymes, which remain intact after PEF treatment. The membrane is a barrier to various hydrophilic mediators^42^, so permeabilized cells can be used in combination with much wider range of mediators providing new insights into cellular machinery. It remains to be elucidated whether hydrophilic mediators could be employed in conjunction with reversible electroporation.

To test how permeabilization affects signal mediated by the lipophilic mediator, we employed menadione. The suggested scheme of charge transfer is the following^42^:

$$ \begin{aligned} & {\text{Menadione}} + {\text{ Quinone}}\,{\text{reductases}} + {\text{ NAD}}\left( {\text{P}} \right){\text{H}} \to {\text{Menadiol}} + {\text{ Quinone reductases}} \\ & \quad + {\text{ NAD}}\left( {\text{P}} \right) ^{-}~{\text{Menadiol}} \to {\text{Menadione}} + { 2} {\text{e}}^{ - } . \\ \end{aligned} $$

When electrodes were covered with untreated cells (E = 0 kV/cm), current of 138 ± 15 nA was detected. After exposure to weak electric fields (E = 4 kV/cm), current slightly decreased down to 117 ± 14 nA. The further rise in electric field strength (E = 10 kV/cm) resulted in even weaker current of 32 ± 15 nA. Exposure to the strongest electric field (16 kV/cm) resulted in further decrease in current down to 23 ± 12 nA.

Menadione can freely cross the cell membrane and enter the cytoplasm where it is then reduced to menadiol by the cytosolic and mitochondrial enzymes catalyzing the electron transfer from NAD(P)H to quinone substrates. It was previously proposed that menadione-mediated current depend on intracellular NAD(P)H content and decrease with its depletion^43^. Intracellular concentrations of NAD(P)H could decrease due to intracellular perturbations like oxidative stress and leakage of these small cofactors. We decided to investigate the latter by measuring fluorescence^27^ of the supernatant from treated (E = 16 kV/cm) and untreated cell suspensions. Specimens were excited with 340 nm light. Fluorescence intensity at 430 nm after pretreatment was eightfold higher. Such results indicate excessive leakage of intracellular compounds. During preparation of electrodes treated cells are transferred onto electrode surface and subsequently into electrochemical cell with higher solution volume, cofactors become highly diluted.

**Figure 6:**
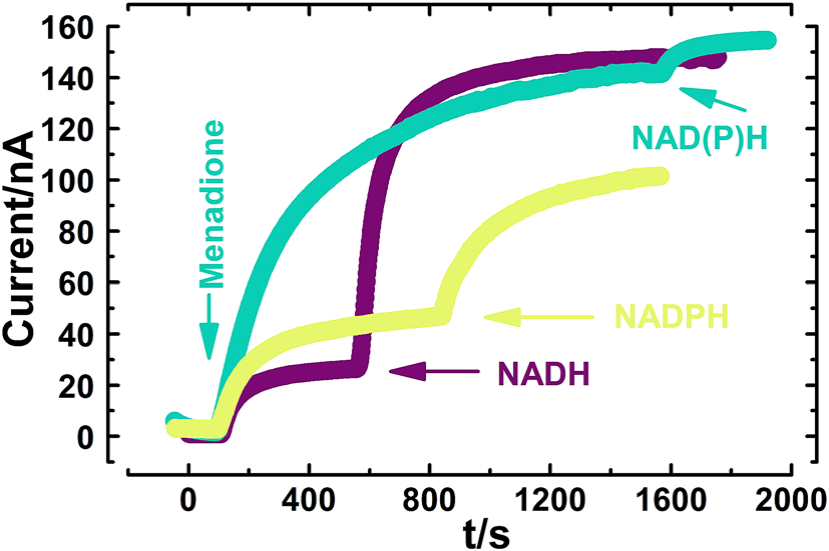
Typical real-time current responses of yeast cells immobilized on carbon paste electrode. Teal curve—untreated cells. Yellow and violet—PEF treated cells (E = 16 kV/cm). Electrolyte composition was 67 μM menadione at an operating potential 0.3 V in phosphate buffer at pH 6. Final concentration of NAD(P)H in the electrolyte was 1 mM.

We performed the further investigation by adding NAD(P)H into the electrolyte during current measurement (Fig. 6). We showed that the decrease in menadione current of PEF treated cells could be complemented entirely by adjusting extracellular concentration of NADH to 1 mM. The addition of NADPH resulted in stronger current as well, but not as high as after addition of NADH. Supplementing untreated cells with NAD(P)H raised current strength by up to 10%. Such findings suggest that the decrease of menadione current is indeed observed due to the leakage of intracellular reducing species through permeable membranes. We, therefore, conclude that formation of hydrophilic pores should not significantly affect the entry of a lipophilic mediator and cause decrease in current strength by itself.

The entire process of exposing cells to redox mediators can be considered as redox stress, which must be counterbalanced by NAD(P)H and the cellular dehydrogenases. Eukaryotic cells use NAD(P)H for multiple purposes, and decrease in its concentration can result in impaired metabolism, calcium homeostasis, gene expression as well as reduction in antioxidative capacity^44^. NADPH is a primary cofactor utilized by cells when reducing artificial mediators^43^. Khlupova et al. showed that membranes of permeabilized and lyophilized cells lose integrity and become NAD(P)H permeable. Such loss of reducing species can be a major cause of why weaker currents are generated^39^. We confirmed that PEF could change permeability leading to the loss of NAD(P)H. Furthermore, we showed that permeabilization could be performed in a controlled way leading to an adjustable signal of the whole-cell sensors.

## Conclusions

**Scheme 1:**
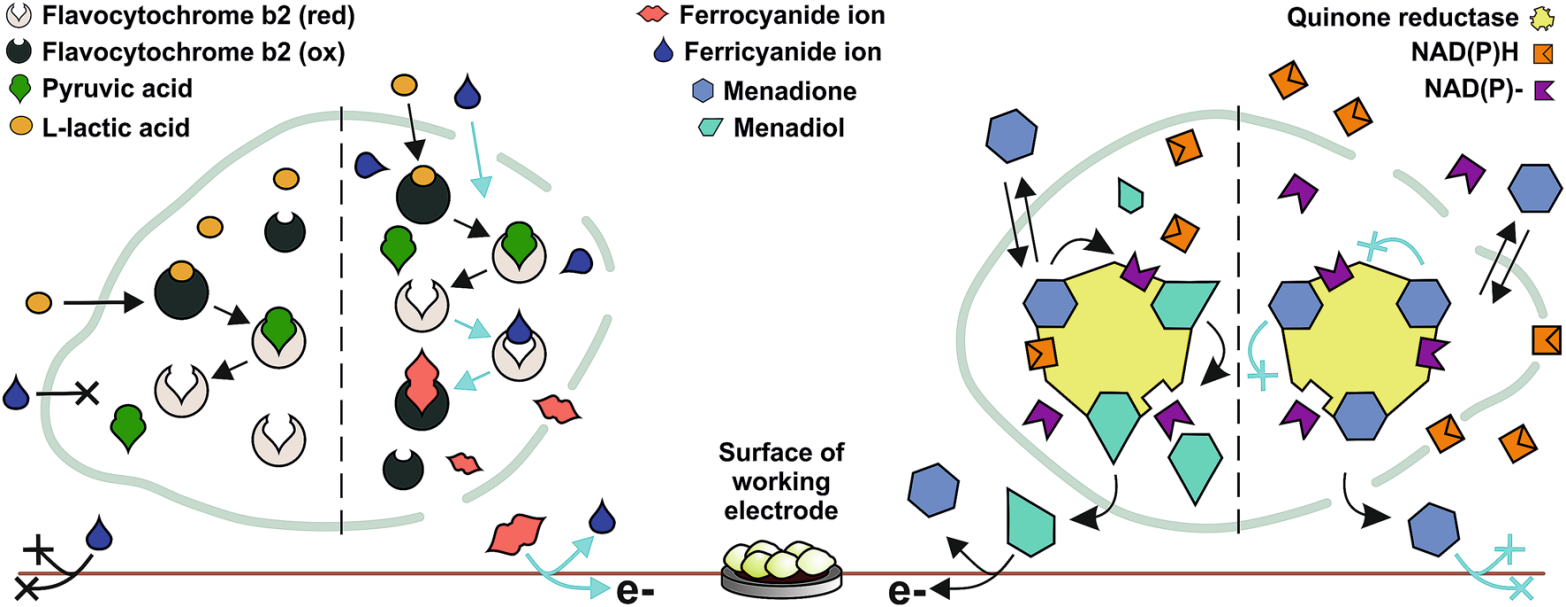
Schematic overview of permeabilization effects on amperometric signal of yeast-modified electrodes.

We showed that after exposure to PEF, the permeability of the cell wall/membrane increased and remained so for at least an hour. Yeast-modified electrode responses to lactic acid were dependent on exposure to PEF and increased with the rise in electric field strength (Scheme 1). Traditional methods for the preparation of yeast cells like lyophilization or chemical treatment (salts, alcohols, acids, detergents) are relatively unspecific and will affect intracellular structures as well as machinery while PEF treatment primarily focuses on the plasma membrane.

PEF treated yeast cells also showed lower redox activity and viability, which decreased with rise in electric field strength. The decrease in menadione current of PEF treated cells could be compensated entirely by adjusting extracellular concentration of NADH to 1 mM. Such results raise attention to the viability kits employing quinone-like mediators. If such mediators are applied to analyze abiotic treatments affecting membrane permeability, they can give ambiguous results. In our study menadione current in PEF treated cells decreased from 100 to ~ 17% while the viability decreased down to ~ 2%, thus highlighting possible inconsistency between methodologies. The complete picture of cellular death, membrane permeability and redox kinetics upon PEF exposure remain to be elucidated.

Our investigation shows that PEF technology can be effectively used to modulate the amperometric signal of yeast-modified electrodes and that they can be used for real-time study of cellular responses to PEF treatment. Such pretreatment can also open the gateways to intracellular enzymes without extraction and allow investigation of both permeability as well as cellular death mechanisms. We conclude that permeabilization in combination with different combinations of growth conditions, inhibitors, mediators, substrates, cofactors will pave the way for the investigation of biochemical pathways through multistep processes.
